# Intrinsic Antioxidant Capacity of Fish Muscle as Potential Link Between Pre-Harvest Biology and *Post Mortem* Flesh Quality in Farmed Fish

**DOI:** 10.3390/antiox15070890

**Published:** 2026-07-18

**Authors:** Cosmas I. Nathanailides, Michael G. Kontominas

**Affiliations:** 1 Department of Agriculture, University of Ioannina, 47100 Arta, Greece; 2 Department of Chemistry, University of Ioannina, 45110 Ioannina, Greece; mkontomi@uoi.gr

**Keywords:** fish muscle, antioxidant capacity, oxidative stress, reactive oxygen species, lipid oxidation, aquaculture

## Abstract

The present review examines markers of intrinsic antioxidant capacity in fish muscle, how these markers change after death, and the point to which their *post mortem* evolution is related to flesh quality and shelf life in farmed fish. Aquaculture parameters such as temperature, salinity and fish feeds can affect intrinsic antioxidant systems throughout the fish life cycle, but, in addition to the effect of aquaculture variables, the redox starting point of muscle at death can be affected by pre-slaughter stressors such as transportation, crowding, fasting, and handling. Based on the results of the studies reviewed in this work, it can be postulated that the rate of *post mortem* lipid and protein oxidation of fish fillets is not determined only by processes occurring after death or during storage, but may also be influenced by the antioxidant status of axial skeletal muscle. This postulation offers an important mechanistic link between (i) pre-slaughter conditions (such as aquaculture production systems, environmental factors, feeding regime, handling stress) and (ii) harvesting methods, with these two determining the oxidative stability of fish fillets via shaping the antioxidant capacity of muscle tissue. This approach highlights the value of linking nutritional, husbandry, harvesting and processing practices in order to improve fish welfare, flesh quality and shelf life.

## 1. Introduction

Reactive oxygen species (ROS) are highly reactive molecules, free radicals and ions that can damage cellular components but also perform vital physiological functions when their generation is precisely regulated [1]. Understanding ROS biology, therefore requires a distinction between oxidative stress and redox signaling [2,3]. To limit oxidative injury while maintaining signaling functions, animals rely on antioxidant systems that include enzymatic defenses, such as superoxide dismutase (SOD), catalase (CAT), glutathione peroxidases (GPx) and glutathione reductase (GR), as well as non-enzymatic scavengers, such as glutathione, ascorbate, tocopherols and carotenoids [4,5]. In fish, antioxidant capacity is not static; it varies among species and type of tissue and is influenced by metabolism, endocrine status, nutritional background and environmental conditions [5,6]. Oxidative stress arises when ROS production exceeds the capacity of antioxidant defenses, leading to loss of homeostasis [7]. This has implications for aquaculture welfare because stressors can trigger compensatory physiological and metabolic responses; when these responses fail, increased ROS production, metabolic dysfunction and cell damage may occur [8,9].

Fish are useful models for redox biology because they occupy diverse aquatic habitats and undergo substantial physiological transitions during development, growth, migration, maturation and reproduction. These transitions modify metabolic demand and can change the balance between ROS production and antioxidant protection. Fish are poikilotherms and can thrive in a range of ecosystems exhibiting seasonal and even diurnal changes in temperature, salinity and oxygen availability.

In natural ecosystems, fish may experience wide fluctuations in environmental parameters such as temperature, salinity and oxygen availability, sometimes approaching the upper or lower limits of their physiological tolerance. Under natural conditions, however, fish can partly respond behaviorally by moving to different depths or nearby habitats, or, in some cases, migrating to more favorable environments. In aquaculture systems, farmed fish are confined within production units and have limited ability to avoid unfavorable environmental or husbandry-related conditions. Consequently, depending on the production system, stocking density, feeding regime and handling practices, farmed fish may experience oxidative challenges that influence tissue antioxidant requirements and redox balance [6,10,11,12]. As a result, redox imbalance may be a significant parameter for farmed fish welfare that is responsible for possible consequences in farmed fish flesh quality. After death, fish flesh deteriorates rapidly because it has a high water activity, near-neutral pH, abundant non-protein nitrogenous compounds, unsaturated fatty acids and active endogenous enzymes. The antioxidant balance of living tissue is progressively lost, allowing lipid and protein oxidation to proceed. Secondary oxidation products generate rancid odors and flavors and contribute to shelf-life reduction [13].

The rate of *post mortem* oxidation may therefore depend partly on the antioxidant status of the muscle at harvest. This status can be modified prior to harvest through diet and husbandry, and after harvest through processing or storage interventions. Exogenous antioxidants, for example, can be added during processing or storage to delay oxidation and preserve fish quality [14,15,16]. Overall, muscle antioxidant capacity reflects the combined effects of growth conditions, pre-harvest management and post-harvest preservation practices, with potential benefits for fish welfare and consumer product quality.

Fish muscle antioxidant capacity is therefore related to both the redox biology and commercial quality of farmed fish. The skeletal muscle redox state varies with intrinsic and extrinsic factors that are also associated with *post mortem* quality traits [17,18,19].

The antioxidant status of several fish tissues may vary with growth trajectory, developmental stage and environmental conditions. This plasticity of antioxidant status is particularly significant for farmed fish, where rapid growth and metabolism are desirable traits for genetic selection, though these fish may exhibit variability of flesh quality [19,20,21]. For example, fish from the same cohort, even when reared under similar conditions, may differ in body composition, lipid profile, muscle structure and post-harvest quality traits [22,23,24,25,26,27,28]. Flesh quality should therefore be viewed not only as a consequence of genetic variability in growth rate or post-harvest fish storage conditions, but also as the outcome of biological and production-related processes that begin during growth and continue throughout harvesting and *post mortem* handling.

These links point to the need to identify the mechanisms that govern the antioxidant phenotype of fish muscle at harvest. This phenotype is shaped *ante mortem* by development, growth, nutrition, environmental exposure and production practices. It may then influence the extent of oxidative and structural deterioration of fish muscle during cold storage [19,29,30].

Although many studies have examined fish responses to oxidative stress and *post mortem* changes in muscle quality, these topics are often considered separately. A more integrated approach is needed to determine how pre-slaughter antioxidant capacity affects the biochemical and structural changes that determine fish flesh quality. *Post mortem* lipid oxidation, enzymatic autolysis and microbial growth are not independent processes, but interacting mechanisms that progressively reduce sensory quality, nutritional value and fish shelf life. Therefore, the antioxidant status of muscle at harvest may represent an early biological determinant of how rapidly these deteriorative pathways proceed during chilled storage.

This mini review proposes that the redox state of fish muscle at harvest is a key link between *ante mortem* biology and *post mortem* flesh quality. It examines how developmental stage, nutrition, environmental exposure and *ante mortem* stress influence muscle antioxidant capacity and, in turn, lipid oxidation, protein oxidation and fish shelf life.

The literature reviewed in this study was identified through a narrative search of the Scopus database on December 2025. Initial searches targeted fish antioxidant status, redox homeostasis, oxidative stress and *post mortem* flesh quality.

**Figure 1:**
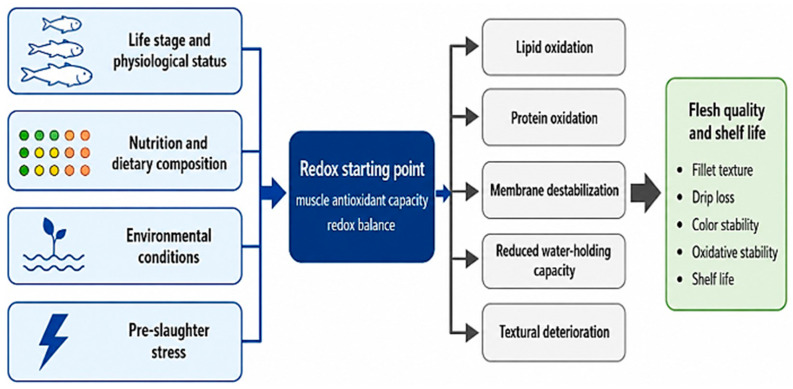
A schematic representation of how pre-slaughter biological and aquaculture-related factors may shape the redox starting point of fish muscle at slaughter and thereby influence *post mortem* deterioration and final flesh quality in farmed fish.

More specifically, during an initial search, the search string fish AND (muscle OR flesh OR fillet) AND antioxidant* was used; this returned 3407 hits. Subsequently, the next search was narrowed by adding the terms *ante mortem* OR *post mortem*, which brought the final total to 37 documents for further review. Published articles were also included in the present review when they were thematically relevant to fish antioxidant biology, aquaculture-related oxidative modulation or flesh quality. Emphasis was placed on recent studies from the past ten years, while older foundational studies were retained when necessary. The present work is a narrative review based on a structured literature search. Its aim was to provide a synthesis of relevant studies on fish oxidative status and implications for *post mortem* flesh quality in farmed fish. Therefore, the findings are discussed as an integrative synthesis of the available literature. Based on this synthesis, Figure 1 summarizes how development, nutrition, environmental conditions and *ante mortem* stress may shape the muscle redox state at harvest and subsequently influence oxidation, membrane stability, texture, flesh quality and fish shelf life.

## 2. *Ante Mortem* Determinants of the Redox Starting Point of Fish Muscle Tissue

Fish antioxidant defense systems include enzymatic and non-enzymatic components from early development. Enzymatic defenses include SOD, CAT, GPx, GR and glutathione S-transferase, whereas non-enzymatic defenses include reduced glutathione and other low-molecular-weight antioxidants. The relative contribution of each component varies with developmental stage, nutritional intake, tissue differentiation and environmental stressors [5,31]. Intrinsic and extrinsic factors therefore interact to shape antioxidant capacity. During embryonic and larval development, antioxidant defense may initially rely on maternal reserves, which are gradually replaced by endogenous production as development progresses and metabolic demand rises [32,33].

After metamorphosis, juvenile fish experience rapid growth and high metabolic turnover. Antioxidant responses differ among species and are affected by growth rate, life mode, diet, temperature and salinity [5,34,35]. During smoltification and gonadal development, muscle metabolism is reorganized and antioxidant enzyme activities can change, including SOD, CAT and GPx activities [20,36]. These changes may represent adaptive redox regulation, while demanding developmental stages can also compromise redox balance [20,37].

Extrinsic factors also influence antioxidant status and oxidative damage in fish tissues. These factors include spatial and seasonal variation in temperature, salinity, oxygen availability, diet, toxin exposure and stocking density [10,18]. Temperature is also a significant parameter; antioxidant responses are strongly tissue-specific and seasonally modulated. For example, in farmed gilthead seabream, the expression of genes encoding SOD, CAT and GR, along with SOD, CAT and GR activities, can vary among white muscle, red muscle, liver and heart depending on different environmental temperature conditions [29]. Among these extrinsic factors, diet is particularly important because feed composition and dietary antioxidants can directly modulate enzymatic antioxidant defenses and oxidative damage in fish tissues. For example, dietary supplementation with *Phyllanthus emblica* fruit, a source of antioxidant compounds [38], significantly enhanced SOD, CAT and GPx activities in tilapia. It also improved immune-related responses and preserved muscle tissue integrity, suggesting better redox homeostasis when used as a feed supplement [39]. Likewise, vitamin E supplementation in tilapia enhanced SOD and CAT activities, lowered malondialdehyde (MDA) in serum and muscle, and improved fillet quality [40]. Similar results were reported in other studies, which showed that dietary inclusion of antioxidants can have a positive effect on *post mortem* antioxidant capacity; for example, dietary vitamin E reduced *post mortem* lipid peroxidation in tilapia muscle [41], while lycopene administered *in vivo* reduced rancidity indices in refrigerated rainbow trout fillets [42]. In European seabass, fermented brewer’s spent grain extracts improved *ante* and *post mortem* oxidative status and reduced muscle lipid peroxidation during refrigerated storage, further supporting the view that diet acts as modulator of the oxidative starting point of fish muscle [43]. In view of the widely reported effects of dietary factors on the antioxidant capacity of fish tissues, it can be concluded that feed composition and functional additives, including plant-derived antioxidants, probiotics, phytogenic and herbal extracts, may modulate antioxidant responses and oxidative resilience in farmed fish [44,45,46,47,48,49].

Endogenous antioxidant protection acts within a pro-oxidant muscle environment that includes iron, heme proteins and pre-existing lipid oxidation products. These factors can accelerate deterioration when antioxidant defenses are insufficient [50]. The effectiveness of intrinsic antioxidant capacity in fish muscle is dynamic and tissue-specific, differs among species and muscle types, and reflects developmental history, nutritional status and environmental exposure [19,51,52]. Table 1 summarizes the main endogenous, exogenous, *ante mortem* and *post mortem* factors that shape intrinsic antioxidant status and flesh quality outcomes, presenting examples of relevant studies that used (i) carnivorous or opportunistic carnivorous species, such as greater amberjack, sea trout, gilt-head seabream, European seabass, meagre, rainbow trout, largemouth bass and large yellow croaker, and (ii) omnivorous or opportunistic omnivorous fish species, such as tilapia and common/mirror carp and (iii) herbivorous or planktivorous species, such as grass carp and silver carp. However, because most of the available evidence concerns farmed fish, trophic classification should be interpreted cautiously. Under aquaculture conditions, fish are usually fed formulated diets that may differ substantially from their natural diets in terms of protein source, lipid composition, antioxidant content and inclusion of functional additives. Therefore, the feeding habit of each species provides useful biological context, but it may not accurately reflect the actual dietary exposure of the fish in the reviewed studies.

Taken together, the studies summarized in Table 1 show that developmental stage, tissue specialization and environmental exposure shape the antioxidant phenotype of fish tissues. Therefore, after harvesting, muscle enters the *post mortem* phase with a redox state already shaped by the biological and production parameters of farmed fish.

## 3. *Ante Mortem* Stress and the Redox Starting Point at Death

Although the antioxidant phenotype of fish muscle is shaped by the biological and production factors discussed above, acute pre-harvest stress may further modify this redox state at death [10,53,60]. This section therefore focuses on harvesting-related stressors and their potential effects on early *post mortem* biochemical changes and flesh quality [61,62].

Pre-harvest stress can activate, deplete or imbalance the intrinsic antioxidant capacity of fish muscle, and stress-related alterations in metabolism, physical traits and organoleptic parameters have been identified as major pathways through which fish stress affects muscle quality [63].

*Post mortem* flesh quality may therefore vary with the stress challenge experienced during transport and slaughtering [62]. Handling stress can increase swimming activity and stress responses in farmed fish, with consequences on fillet muscle quality. Escape activity, anaerobic metabolism, depletion of muscle energy stores, lactate accumulation and faster pH decline can accelerate *rigor mortis* and *post mortem* deterioration. In European sea bass, increased handling stress prior to harvesting was associated with faster pH decline, earlier *rigor mortis*, increased drip loss and lipid oxidation during storage, indicating effects on both welfare and flesh quality [61].

Aquaculture production conditions can affect the stress-related consequences on fish flesh. Hematyar et al. [63] observed that pre-slaughter anoxia and higher stocking density in largemouth bass were associated with lower initial pH, earlier onset of *rigor mortis*, increased antioxidant enzyme activity, higher lipid oxidation, and reduced fillet hardness; however, within the anoxia treatment, fish reared at the lowest stocking density showed higher initial hardness than those reared at higher fish density. These observations indicate that reducing handling stress may improve both fish welfare and the *post mortem* flesh quality changes in fish muscle. Another example of this interaction was recently reported by Martinez Villalba et al. [64] for farmed rainbow trout, where pre-slaughter fasting and seasonal thermal conditions modified muscle glycogen content and affected pH, rigor development and color during the first 24 h *post mortem* [65]. Based on the above examples, it can be argued that the effect of pre-harvest management on flesh quality is not fixed but depends on the physiological and environmental condition of the fish at harvest. The initial *post mortem* antioxidant or oxidative marker value should therefore not be interpreted as a simple *ante mortem* baseline. It is more appropriately viewed as an early redox state of the muscle, shaped both by the physiological condition developed during rearing and by the acute stress imposed during harvesting, handling, stunning and killing. Studies comparing stunning or slaughter methods show that acute pre-slaughter stress can affect cortisol and glucose responses, pH decline, ATP degradation, antioxidant enzyme activities, lipid oxidation, protein carbonylation and texture changes during storage [9,54,55,56,65,66,67]. There are several parameters related to the above. For example, growth phenotype can affect total antioxidant capacity and SOD activity in meagre fillets during cold storage, and although dietary essential oil supplementation in trout did not prevent the immediate response to slaughter stress, it did delay oxidative deterioration during frozen storage [19,56]. Thus, the redox state measured immediately after slaughter should be considered as the combined outcome of rearing history and harvest/slaughter stress. The later development of oxidative damage during storage probably depends both on the severity of the acute stressor at death and on the remaining antioxidant capacity of the muscle.

## 4. From Redox Homeostasis to Early *Post Mortem* Lipid-Mediated Flesh Deterioration

As illustrated in Figure 1, this early redox state provides the biochemical background against which *post mortem* deterioration develops. After death, the progressive loss of ATP, ionic and membrane homeostasis, antioxidant buffering and mitochondrial integrity promote lipid and protein oxidation and structural weakening of the tissue, a process which is enhanced as antioxidant capacity declines. During this early period, ATP depletion, pH decline, mitochondrial dysfunction, reactive oxygen species formation, loss of antioxidant buffering and protein oxidation may contribute to freshness loss and texture deterioration [64,67,68,69]. This is especially relevant for fish muscle because of its high unsaturated lipid content and structural fragility compared to terrestrial animal muscle [19,30]. Early antioxidant decline allows oxidative reactions to spread, and this process is influenced by muscle structure and mitochondrial distribution. Mitochondrial density differs among species and tissues; red and white muscle may also differ in response to thermal acclimation [70]. Mitochondria are among the first organelles affected during *post mortem* change. Loss of mitochondrial integrity can increase ROS production, reduce antioxidant defense and contribute to myofibrillar protein degradation, texture loss and declining water-holding capacity [64]. The extent of oxidative injury may also vary with membrane lipid composition and mitochondrial density. Reactive species differ in their ability to cross membranes; some move relatively readily, whereas others remain compartmentalized because of lower membrane permeability and rapid intracellular consumption [58]. Oxidation of membrane polyunsaturated fatty acids (PUFAs) can therefore initiate chain reactions that damage membranes and associated proteins, contributing to structural weakening of the fish muscle, i.e., the fillet [58,71].

Lipid and protein oxidation interact during deterioration: lipid oxidation products can damage muscle proteins, while oxidative modification of membranes and myofibrillar proteins such as myosin and actin contribute to structural weakening [30,51,71,72,73,74].

Across species and aquaculture production systems, fish muscle shows considerable variation in flesh quality traits, which can reflect genetic and environmentally induced variability and their interaction [19,72]. This variation can also influence the pattern of *post mortem* quality change.

## 5. Significance of Redox Homeostasis for Post Mortem Changes in Fish Fillet Quality and Shelf Life

Intrinsic antioxidant capacity may influence product shelf life by providing an early defense against *post mortem* lipid oxidation, protein oxidation and membrane destabilization. The degree of protection varies with species, muscle composition and storage conditions [19,30,50]. Post mortem lipid oxidation is of particular importance because it reduces sensory quality and can lower nutritional value. Although long-chain omega-3 polyunsaturated fatty acids are beneficial for human health, their high degree of unsaturation makes fish muscle particularly vulnerable to oxidative degradation and rancidity compared to terrestrial meat, which contains a higher proportion of saturated fats [23,75,76,77,78]. This lipid susceptibility has direct consequences on fillet quality, since secondary lipid oxidation products can rapidly reduce quality through off-odors, off-flavors and structural deterioration during cold storage [13,59,79].

Dietary supplementation represents one of the main pre-harvest approaches used to modify antioxidant status. Table 2 provides some examples of the main effects of antioxidant feed compounds reported to have effects on antioxidant responses and post mortem flesh quality variables. These studies indicate that dietary bioactive compounds may improve antioxidant status prior to harvest and, in some cases, reduce lipid peroxidation, rancidity development or oxidative deterioration during storage. However, the post mortem variables evaluated differ among studies, which makes direct comparison difficult.

Final quality is also shaped by the interaction between muscle fiber structure, lipid composition and antioxidant status [59,80]. Muscle cellularity, including myofiber size and density and the balance between hypertrophic and hyperplastic growth, can influence texture and water retention depending on species and production system [59,65,81,82]. Oxidative modification of membrane and myofibrillar proteins, including myosin and actin, can reduce water-holding capacity and promote undesirable changes in texture [30,73,74].

In addition to pre-harvest dietary modulation, post-harvest packaging technologies, including active and intelligent packaging, may also influence oxidative deterioration during storage. For this reason, the shelf life of farmed fish fillets could be a result of the combined outcome of muscle redox status at harvest, remaining intrinsic antioxidant protection and the packaging or preservation strategy used during storage [13,14,30].

The potential significance of antioxidant status for final product quality is illustrated by the studies summarized in Table 3, which provide some examples of how feeding regimes can enhance intrinsic antioxidant capacity, which in turn may constitute a defense mechanism against *post mortem* flesh quality deterioration [83].

## 6. Methodological Challenges in Linking Fish Redox Status with Flesh Quality Traits

Both redox biology and flesh quality are influenced by multiple intrinsic and extrinsic factors in aquacultured fish. Comparisons among studies are difficult because species, tissues, assay protocols, reporting units and normalization methods often differ [5,86]. Experimental designs should also distinguish normal physiological variation from stress-related redox imbalance. Enzymatic markers such as SOD, CAT, GPx and GR and non-enzymatic markers such as glutathione, tocopherols and total antioxidant capacity reflect different aspects of redox buffering. They may respond differently to environmental or developmental factors, so that limited marker sets may misrepresent antioxidant status [87].

An increase in the activity of antioxidant enzymes can indicate a compensatory response, for example, due to oxidative stress under a given physiological context [46,53]. It should be stated, however, that the interpretation of any changes in the activity of antioxidant defense mechanisms requires evidence-based evaluation. In this direction, it would be helpful to integrate biochemical antioxidant data with structural and product-level measures such as lipid oxidation, protein oxidation, drip loss and flesh texture; this may clarify the mechanisms linking redox status to flesh quality [19,30,46,86].

Future research should examine how life history, environment, tissue physiology and flesh quality interact through redox status. Longitudinal studies that monitor antioxidant and quality markers over time could help distinguish adaptive responses from progressive deterioration [29,32,37]. Studies of subcellular organization and ROS compartmentalization could provide more mechanistic insight into how oxidative reactions develop within fish muscle [58,70]. Redox status should also be integrated into aquaculture and post-harvest management. Optimizing pre-slaughter conditions and connecting oxidative physiology to farm-to-product management may be more effective than focusing only on storage [47,65]. Comparative studies across species and environments, supported by advanced omics and physiological tools, can further clarify the redox mechanisms that shape fish quality [6,18].

## 7. Conclusions

The studies reviewed in this work support the view that the antioxidant status of farmed fish muscle at harvest is shaped by several parameters, including aquaculture production system, developmental stage, growth rate, nutrition, environment, and harvest-related stress. These *in vivo* variables can affect critical *post mortem* quality traits, including lipid/protein oxidation, water-holding capacity, texture, and shelf life. Thus, *ante mortem* and *post mortem* handling and fish preservation conditions should not be viewed as separate quality-control steps, but as consecutive parts of the same aquaculture production chain, linking (*i*) fish nutrition, (*ii*) husbandry and (*iii*) harvesting practices to the intrinsic antioxidant capacity of fish muscle tissue and *post mortem* flesh quality of farmed fish fillets.

This approach may help elucidate the biological mechanisms through which pre-harvest conditions influence post-harvest oxidative stability, texture development, water-holding capacity and shelf life, thereby providing a more integrated framework for improving the quality and shelf life of farmed fish fillets.

## Figures and Tables

**Table 1:** Main parameters affecting intrinsic antioxidant status and *post mortem* fish muscle quality of farmed fish.

Factors	Main Determinants	Consequence for Muscle	Expected *Post Mortem* Implication	References
Endogenous	Development, growth, maturation, aging	Shifts in antioxidant systems and metabolism	Different oxidative starting point at harvest	[5,6,20,37]
Exogenous	Temperature, salinity, nutrition, density, hypoxia	Modulation of redox phenotype and tissue composition	Altered resistance to oxidation and structural damage	[11,18,29,53,54]
Pre-harvest and harvesting phase	Transport, crowding, fasting, handling, grading, harvesting	Stress-related energy depletion, altered antioxidant response, increased protein oxidation	Faster ATP degradation, protein carbonylation, texture loss and reduced oxidative stability during storage	[55,56,57]
Early *post mortem* phase	ATP loss, membrane instability, ROS compartmentation	Declining antioxidant buffering and rising lipid/protein oxidation	Texture loss, drip loss, reduced WHC, color change	[19,58,59]
*Post mortem* quality changes	Cold storage conditions, exposure to oxygen	Lipid and protein oxidation	Shorter shelf life and lower market quality	[30,50,52]

WHC = water-holding capacity.

**Table 2:** Examples of dietary antioxidant compounds used for enhancing the antioxidant capacity of fish flesh and the reported effects.

Dietary Antioxidant Compound Used	Fish Species, Main Feeding Habits	Flesh Quality Variables Evaluated	Main Reported Effects	Reference
*Phyllanthus emblica* fruit	Red tilapia, omnivorous	Antioxidant enzyme activity, immune response, muscle tissue integrity	Increased SOD, CAT and GPx activities and improved *post mortem* muscle tissue integrity during refrigerated storage	[37,38]
Vitamin E	Tilapia, omnivorous	Antioxidant capacity, MDA, fillet quality/textural traits	Increased SOD and CAT activities, reduced MDA in serum and muscle, and improved *post mortem* fillet quality during refrigerated storage	[39]
Vitamin E	Hybrid tilapia, omnivorous	Post mortem lipid peroxidation in muscle	Reduced lipid peroxidation in *post mortem* muscle tissue during refrigerated storage	[40]
Lycopene	Rainbow trout, carnivorous	Rancidity indices during refrigerated storage	Reduced rancidity development in fillets during refrigerated storage	[41]
Fermented brewer’s spent grain extract	European seabass, carnivorous	*Ante* and *post mortem* oxidative status, muscle lipid peroxidation during refrigerated storage	Improved oxidative status and reduced muscle lipid peroxidation during refrigerated storage	[42]

**Table 3:** *Post mortem* flesh quality traits associated with antioxidant phenotype and oxidative deterioration in fish muscle.

Quality Trait	Main Underlying Mechanisms	Why Antioxidant Status Matters	References
Drip loss/WHC	Membrane damage, protein denaturation, structural weakening	Lower antioxidant buffering may accelerate membrane and protein oxidation	[19,61,84]
Texture/firmness	Myofibrillar degradation, proteolysis, altered muscle architecture, protein carbonylation of enzymes and muscle structural proteins	Redox failure may intensify softening and structural instability	[19,55,56,59]
Color/appearance	Lipid oxidation, pigment-related secondary reactions, surface water loss	Better antioxidant status may delay visible loss of freshness	[30,50,51,79,83,85]
Shelf life	Combined lipid oxidation, protein oxidation, exudation, structural collapse	Higher intrinsic antioxidant capacity may slow overall deterioration	[19,30,56,59]

WHC = water-holding capacity.

## Data Availability

No new data were created or analyzed in this study. Data sharing is not applicable to this article.
